# Determination of the minimum protective dose of a glycoprotein-G-deficient infectious laryngotracheitis virus vaccine delivered via eye-drop to week-old chickens

**DOI:** 10.1371/journal.pone.0207611

**Published:** 2018-12-06

**Authors:** Mesula G. Korsa, Joanne M. Devlin, Carol A. Hartley, Glenn F. Browning, Mauricio J. C. Coppo, José A. Quinteros, Carlos A. Loncoman, Adepeju E. Onasanya, Dulari Thilakarathne, Andrés Diaz-Méndez

**Affiliations:** Asia-Pacific Centre for Animal Health, Melbourne Veterinary School, Faculty of Veterinary and Agricultural Sciences, The University of Melbourne, Parkville, Victoria, Australia; Sun Yat-Sen University, CHINA

## Abstract

Infectious laryngotracheitis (ILT) is an upper respiratory tract disease of chickens that is caused by infectious laryngotracheitis virus (ILTV), an alphaherpesvirus. This disease causes significant economic loses in poultry industries worldwide. Despite widespread use of commercial live attenuated vaccines, many poultry industries continue to experience outbreaks of disease caused by ILTV. Efforts to improve the control of this disease have resulted in the generation of new vaccine candidates, including ILTV mutants deficient in virulence factors. A glycoprotein G deletion mutant vaccine strain of ILTV (ΔgG ILTV), recently licenced as Vaxsafe ILT (Bioproperties Pty Ltd), has been extensively characterised *in vitro* and *in vivo*, but the minimum effective dose required to protect inoculated animals has not been determined. This study performed a vaccination and challenge experiment to determine the minimum dose of ΔgG ILTV that, when delivered by eye-drop to seven-day-old specific pathogen-free chickens, would protect the birds from a robust challenge with a virulent field strain of virus (class 9 ILTV). A dose of 10^3.8^ plaque forming units was the lowest dose capable of providing a high level of protection against challenge, as measured by clinical signs of disease, tracheal pathology and virus replication after challenge. This study has shown that the ΔgG ILTV vaccine strain is capable of inducing a high level of protection against a virulent field virus at a commercially feasible dose. These results lay the foundations upon which a commercial vaccine can be developed, thereby offering the potential to provide producers with another important tool to help control ILTV.

## 1. Introduction

Infectious laryngotracheitis virus (ILTV) is an alphaherpesvirus that causes acute upper respiratory tract disease in chickens and has significant economic importance for poultry industries throughout the world [1]. Commercially available attenuated vaccines are commonly used to help control the disease [2, 3]. Despite the use of these vaccines, many poultry industries continue to experience outbreaks of disease caused by ILTV. In Australia, natural recombination between two distinct live attenuated vaccine strains resulted in the generation of virulent recombinant field strains of ILTV, including the class 8 and 9 viruses [4]. These recombinant viruses have spread and have caused disease in major poultry-producing areas of Australia. Another recombinant field virus, class 10 ILTV, emerged in 2013 in Australian poultry and has also spread and caused outbreaks of disease [5]. Taken together, available data suggest that improvements to control strategies for ILTV are needed, including tools to control the recently emerged, virulent recombinant viruses.

Recently, efforts to improve the use of ILTV vaccines, and to develop new ILTV vaccines, have resulted in the availability of more options for control of the disease by poultry producers. In some countries, vectored ILTV vaccines are in widespread use. These vaccines use a virus vector, such as fowlpox virus or herpesvirus of turkeys, to deliver specific ILTV antigens [6–11]. The generation of ILTV mutants deficient in virulence factors (deletion mutant vaccines) has also been investigated and these have the potential to offer additional tools for disease control [2, 3]. Previous *in vivo* and *in vitro* studies have shown that ILTV deficient in the virulence factor glycoprotein G (gG, a chemokine binding protein) has characteristics that would make it suitable for use as an attenuated vaccine (ΔgG ILTV) [12–20]

The ΔgG ILTV vaccine strain has been extensively studied *in vivo*. The first study to investigate the potential efficacy of the ΔgG ILTV vaccine strain delivered the virus at a dose of 10^3^ plaque forming units (PFU), via intratracheal inoculation to four-week-old specific pathogen free (SPF) chickens, followed by challenge of the birds with the CSW-1 field strain (class 4) of ILTV at seven weeks of age. Birds that received the ΔgG ILTV vaccine had significantly fewer clinical signs than unvaccinated birds [13]. The suitability of the ΔgG ILTV vaccine strain was then investigated using delivery methods suitable for mass vaccination programs, including delivery via drinking water, eye-drop or *in ovo* inoculation [12, 15–17]. The results from these studies demonstrated that the ΔgG ILTV vaccine strain has desirable safety and efficacy characteristics. Although this vaccine strain has shown promise as a potential alternative to conventionally attenuated live ILTV vaccines, the minimum effective dose required to protect inoculated birds has not been determined. The aim of this study was therefore to determine the minimum dose of ΔgG ILTV that, when delivered by eye-drop to seven-day-old SPF chickens, would protect the birds from a robust challenge with virulent, recombinant class 9 virus.

## 2. Materials and methods

### 2.1. Experimental design, animals and virus strains used in this study

The study was approved by the Animal Ethics Committee of the Faculty of Veterinary and Agricultural Sciences, The University of Melbourne (Animal Ethics ID -1413097) in accordance with institutional and national guidelines. After hatching, 121 SPF chicks were divided into six groups (five groups with 22 birds and one group with 11 birds) that were each housed in separate isolator units. Vaccine (Vaxsafe ILT) was obtained from the manufacturer (Bioproperties Pty Ltd) as freeze-dried product, five passages beyond the master seed. Inoculum containing the ΔgG ILTV vaccine strain was then prepared by re-suspending the freeze-dried product in commercial sterile diluent (Merial Select Inc.) according to the manufacturer’s directions. At seven days of age, four groups, each of which contained 22 birds, were vaccinated with 10^5.0^ PFU, 10^3.8^ PFU, 10^3.5^ PFU, or 10^3.2^ PFU of the ΔgG ILTV vaccine strain via eye-drop in a 30 μL volume. All ILTV titres were determined using PFU assays performed in LMH cells, as previously described [12]. The remaining two groups of birds were mock vaccinated by administering the same volume of sterile diluent. After mock-vaccination, one bird in the group of 11 birds developed a leg injury (unrelated to mock-vaccination) and was euthanized for animal welfare reasons, consistent with Animal Ethics ID -1413097.

At 28 days of age the vaccinated groups of birds were challenged with class 9 ILTV, a virulent recombinant field strain (10^3.0^ PFU/bird), resuspended in vaccine diluent, with approximately half of the dose (10^2.7^ PFU) delivered onto the conjunctiva (40 μL each eye) and the other half (10^2.7^ PFU) into the trachea (150 μL). Similar procedures were used to inoculate one of the unvaccinated groups of 22 birds with the same virulent field strain, as a positive control group (unvaccinated-challenged). The remaining unvaccinated group of 11 birds was kept as a negative control group (unvaccinated-unchallenged). Birds were scored for clinical signs of disease from three to six days post-challenge (dpc). Typical clinical signs due to challenge with wild type virus are expected to peak during this period [12]. Any birds that showed severe signs of disease were humanely euthanized by anaesthetic (halothane) overdose and these birds were included in the recorded mortalities. Any recorded severe clinical signs in these birds prior to being euthanised were also included in the cumulative clinical scores. All remaining birds were euthanized at 7 dpc. At necropsy, the severity of ILTV induced tracheal pathology was recorded [12], and conjunctival and tracheal swabs were collected and placed in 1 mL of sterile viral transport medium consisting of Dulbecco’s minimal essential medium (DMEM, Sigma-Aldrich) supplemented with 10% v/v foetal bovine serum (Sigma-Aldrich), 10 mM HEPES, pH 7.6, 50 μg/ml ampicillin (Sigma-Aldrich) and 2.5 μg/ml amphotericin B (Astral Scientific). The samples were transported on ice and then immediately stored at -80°C until processing. Clinical scores, gross pathological lesions, virus detection and replication in the conjunctival and tracheal mucosa were used to assess the level of protection induced in response to the different doses of ΔgG ILTV and are described in further detail below.

### 2.2. Clinical scoring

Clinical signs of demeanour, dyspnoea, and conjunctivitis were scored as previously described [13]. Briefly, demeanour was scored as 0 (normal demeanour), 1 (depressed demeanour) or 2 (severely depressed demeanour). Similarly, conjunctivitis was scored as 0 (conjunctival mucosa normal), 1 (partial eye closure) or 2 (compete eye closure, marked conjunctivitis), and dyspnoea was scored as 0 (normal breathing), 1 (mild dyspnoea), 2 (moderate dyspnoea), 3 (marked dyspnoea), or 4 (severe gasping).

### 2.3 Gross tracheal pathology scoring

Gross tracheal pathology was scored as previously described [13]. Briefly, at necropsy, the trachea was removed and then cut lengthways for gross lesion scoring. The severity of the lesions was scored as 0 (normal),1 (mild amount of mucus present), 2 (moderate amount of mucous present), 3 (large amount of mucus present, some blood also present, or diphtheritic material present but not appearing to block the trachea) or 4 (large amount of mucus present, significant blood also presents, or a diphtheritic plug present and blocking the trachea).

### 2.4 Virus detection and quantification

DNA was extracted from the medium containing the conjunctival and tracheal swabs that were collected during necropsy using the KingFisher Flex Purification System (ThermoFisher Scientific) following the manufacturer’s instructions. Positive extraction control samples (diluted stocks of the SA2 ILTV vaccine strain; Zoetis) and negative extraction control samples (distilled water) were included in each extraction plate. Ninety microliter samples of eluted DNA were sealed and stored at -20°C until the extracts were tested for the presence of ILTV DNA by real-time quantitative PCR using oligonucleotide primer pairs that amplify a 113 bp region of the UL15 gene of ILTV, as described previously [21]. To generate a standard curve, a 10-fold dilution series of the UL15 sequence cloned into pGEM-T (Promega) was prepared using a QIAgility robot (Qiagen) and included in triplicate in each run to enable quantitation of the ILTV genome concentration in each of the extracted samples. Only samples that produced amplicons with a melt curve that matched those of the standard curve samples were regarded as positive for the presence of ILTV DNA. Calculations of the concentration of the ILTV genome in the extracted samples were performed using Rotorgene Q version 2.1.0 (Qiagen) and the concentration in viral genome copy numbers per reaction were log10 transformed for statistical analysis, with the lower limit of detection for the assay defined as 100 genome copies per reaction.

### 2.5 Statistical analyses

GraphPad Prism 6 (GraphPad Software) and Excel 2016 (Microsoft) were used for data analyses. Mann-Whitney tests were used to compare differences between the groups in the scores for clinical signs and gross tracheal pathology. A one-way analysis of variance, in conjunction with Dunnett’s Multiple Comparisons test, was used to compare differences between the groups in viral genome concentrations. Fisher’s Exact Test was used to compare differences between the groups in the proportions of birds positive for ILTV by qPCR and in mortality rates.

## 3. Results

Mortality rates, the severity of virus-induced clinical signs of disease (demeanour, conjunctivitis and dyspnoea), the severity of gross tracheal pathology, and the level of viral replication in tracheal and conjunctival mucosa were assessed after challenge. The vaccinated and control groups were compared in order to identify a suitable minimum effective dose of the vaccine.

### 3.1 Clinical signs of disease

Scores for demeanour, conjunctivitis and dyspnoea are summarised in Tables 1, 2 and 3, respectively. For all these parameters, the highest scores were seen in the positive control (unvaccinated-challenged) group, and the scores in the negative control (unvaccinated-unchallenged) group were all zero. The differences between the negative control and the positive control group were significant for all parameters. There were no significant differences between the group that received the highest dose of vaccine (10^5.0^ PFU) and the negative control group at any time point for any of these parameters. This was also true for the group that received the second highest dose of vaccine (10^3.8^ PFU), although when the scores were summed to yield cumulative scores, this group had a cumulative conjunctivitis score that was significantly higher than the cumulative score for the negative control group (Table 2). The scores for the groups of birds that received the lower doses of vaccine (10^3.2^ or 10^3.5^ PFU) were significantly higher than those for the negative control group for all disease parameters on at least two of the four time points. In these groups, the cumulative scores for all parameters were significantly higher than those of the negative control group (Tables 1–3).

**Table 1:** Scores for demeanour on days 3 to 6 after challenge in unvaccinated birds and in birds vaccinated with different doses of the ΔgG ILTV vaccine administered via eye-drop.

Vaccine dose (PFU)	Challenge ^‡^	Median demeanour score (range)*				
		3 dpc^	4 dpc	5 dpc	6 dpc	Cumulative^†^
None	None	0 (0–0) ^A^	0 (0–0) ^A^	0 (0–0) ^A^	0 (0–0) ^A^	0 (0–0) ^A^
None	Class 9^‡^	1 (0–2) ^B^	1 (1–2) ^B^	1 (1–2) ^B^	1 (1–1) ^B^	3 (0–5) ^B^
ΔgG ILTV (10^5.0^)	Class 9	0 (0–0) ^A^	0 (0–0) ^A^	0 (0–0) ^A^	0 (0–0) ^A^	0 (0–0) ^A^
ΔgG ILTV (10^3.8^)	Class 9	0 (0–1) ^A^	0 (0–1) ^A^	0 (0–1) ^A^	0 (0–1) ^A^	0 (0–3) ^A^
ΔgG ILTV (10^3.5^)	Class 9	0 (0–1) ^A^	0 (0–2) ^C^	1 (0–2) ^C^	0 (0–1) ^A^	1 (0–5) ^C^
ΔgG ILTV (10^3.2^)	Class 9	0 (0–1) ^A^	0 (0–1) ^A, C^	1 (0–2) ^C^	0 (0–1) ^A^	1 (0–4) ^C^

*Values labelled with the same uppercase superscript letter in the same column were not significantly different (P > 0.05, Mann-Whitney test)

^‡^ Class 9 challenge = 10^3^ PFU of class 9 ILTV delivered via eye-drop and intra-tracheal inoculation

^ dpc = days post-challenge

^†^ Cumulative = sum of scores on all days for each individual bird

**Table 2:** Scores for conjunctivitis on days 3 to 6 after challenge in unvaccinated birds and in birds vaccinated with different doses of ΔgG ILTV vaccine administered via eye-drop.

Vaccine dose (PFU)	Challenge^‡^	Median conjunctivitis score (range)*				
		3 dpc^	4 dpc	5 dpc	6 dpc	Cumulative^†^
None	None	0 (0–0) ^A^	0 (0–0) ^A^	0 (0–0) ^A^	0 (0–0) ^A^	0 (0–0) ^A^
None	Class 9^‡^	0 (0–1) ^B^	1 (0–2) ^B^	1 (0–2) ^B^	1 (0–1) ^B^	1 (0–6) ^B^
ΔgG ILTV (10^5.0^)	Class 9	0 (0–0) ^A^	0 (0–0) ^A^	0 (0–0) ^A^	0 (0–0) ^A^	0 (0–0) ^A, C^
ΔgG ILTV (10^3.8^)	Class 9	0 (0–0) ^A^	0 (0–0) ^A^	0 (0–2) ^A^	0 (0–0) ^A^	0 (0–2) ^C^
ΔgG ILTV (10^3.5^)	Class 9	0 (0–1) ^A^	0 (0–1) ^A, C^	0 (0–2) ^A^	0 (0–2) ^C^	0 (0–5) ^D^
ΔgG ILTV (10^3.2^)	Class 9	0 (0–1) ^B^	0 (0–2) ^C^	0 (0–1) ^A^	0 (0–1) ^B, C^	0 (0–4) ^D^

*Values labelled with the same uppercase superscript letter in the same column were not significantly different (P > 0.05, Mann-Whitney test)

^‡^ Class 9 challenge = 10^3^ PFU of class 9 ILTV delivered via eye-drop and intra-tracheal inoculation

^ dpc = days post-challenge

^†^ Cumulative = sum of scores on all days for each individual bird

**Table 3:** Scores for dyspnoea on days 3 to 6 after challenge in unvaccinated birds and in birds vaccinated with different doses of ΔgG ILTV vaccine administered via eye-drop.

Vaccine dose (PFU)	Challenge^‡^	Median dyspnoea score (range)*				
		3 d.p.c^	4 d.p.c	5 d.p.c	6 d.p.c	Cumulative^†^
None	None	0 (0–0) ^A^	0 (0–0) ^A^	0 (0–0) ^A^	0 (0–0) ^A^	0 (0–0) ^A^
None	Class 9^‡^	1 (0–3) ^B^	1 (0–3) ^B^	1 (0–2) ^B^	0 (0–1) ^B^	2 (1–6) ^B^
ΔgG ILTV (10^5.0^)	Class 9	0 (0–0) ^A^	0 (0–0) ^A^	0 (0–0) ^A^	0(0–0) ^A^	0 (0–0) ^A^
ΔgG ILTV (10^3.8^)	Class 9	0 (0–1) ^A^	0 (0–1) ^A^	0 (0–2) ^A^	0(0–1) ^A^	0 (0–4) ^A, C^
ΔgG ILTV (10^3.5^)	Class 9	0 (0–1) ^A^	0 (0–2) ^A^	0 (0–3) ^B^	0(0–1) ^B^	1 (0–4) ^C^
ΔgG ILTV (10^3.2^)	Class 9	0 (0–1) ^B^	1 (0–4) ^B^	1 (0–2) ^B^	0(0–1) ^B^	2 (0–5) ^B^

*Values labelled with the same uppercase superscript letter in the same column were not significantly different (P > 0.05, Mann Whitney test)

^‡^ Class 9 challenge = 10^3^ PFU of class 9 ILTV delivered via eye-drop and intra-tracheal inoculation

^ dpc = days post-challenge

^†^ Cumulative = sum of scores on all days for each individual bird

### 3.2 Viral detection and quantification in tracheal and conjunctival swabs

**Fig 1:**
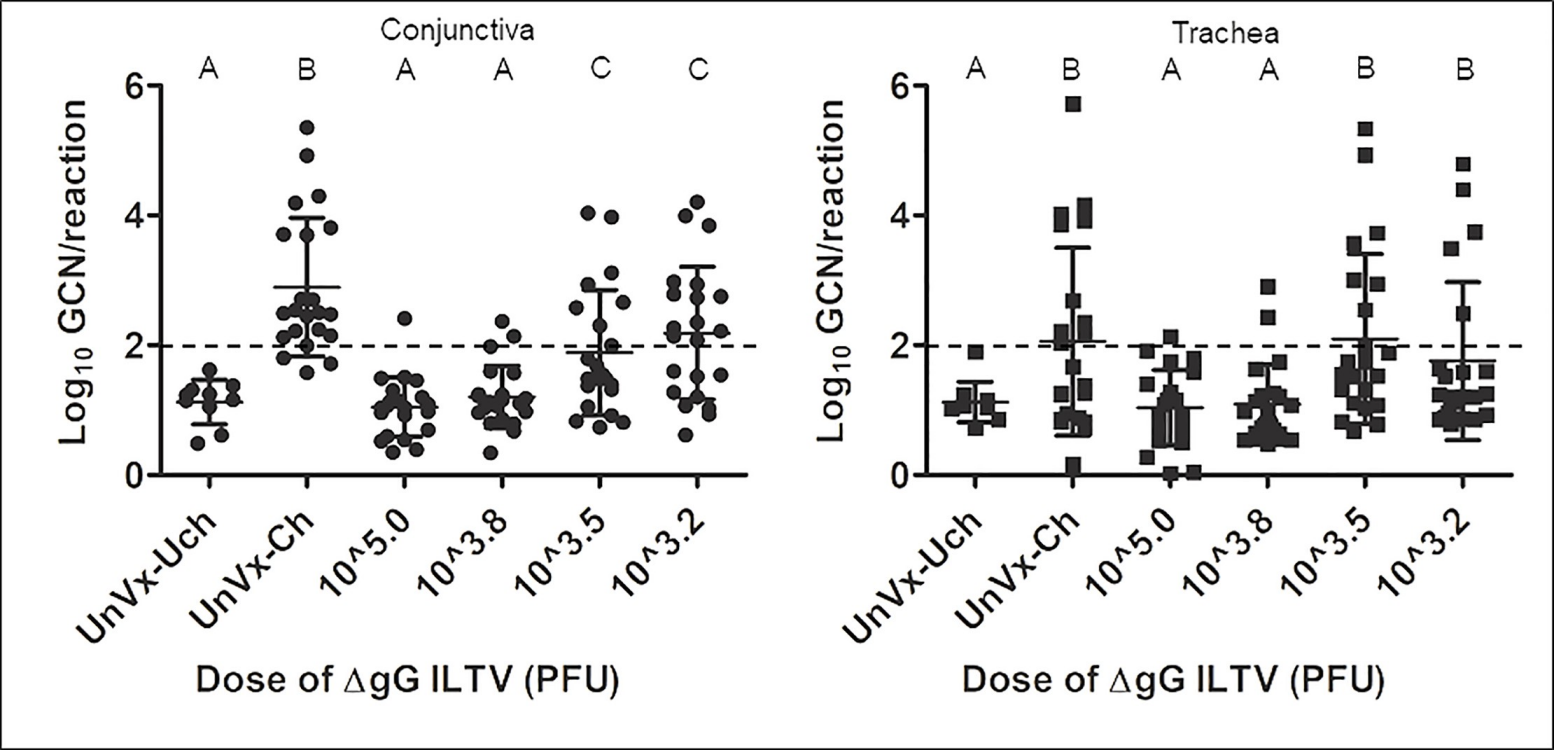
Scatter plot of concentrations of ILTV as determined by qPCR on extracts from conjunctival or tracheal swabs of unvaccinated birds and birds vaccinated with different doses of ΔgG ILTV at day 7 after challenge with class 9 ILTV. Values labelled with the same upper-case letter (A, B, C) were not significantly different (P > 0.05, one-way analyses of variance in conjunction with Dunnett’s Multiple Comparisons test) in each panel (conjunctiva or trachea). GCN = genome copy number, UnVx-Uch = unvaccinated and unchallenged, UnVx-Ch = unvaccinated and challenged, PFU = plaque forming units. Means (horizontal solid lines) and standard deviations (error bars) are also shown. The cut off value for positivity was 100 genome copies per reaction and this is indicated by a horizontal dashed line.

The results for virus quantification in tracheal and conjunctival swabs after challenge are presented in Fig 1. The groups vaccinated with the two higher doses of the vaccine (10^3.8^ PFU or 10^5.0^ PFU) had concentrations of virus at both sites that were significantly lower than those of birds in the positive control group, but that were not significantly different from those of the negative control (unvaccinated-unchallenged) birds. The groups vaccinated with the two lower doses of vaccine had concentrations of virus at both sites that were significantly higher than those of negative control birds. There was no significant difference in virus concentrations in the trachea of the birds that received the two lower doses of vaccine and that of birds in the positive control group, but the two lower doses of the vaccine were partially protective in the conjunctiva, resulting in significantly lower concentrations of virus at this site than in the positive control birds (Fig 1).

**Fig 2:**
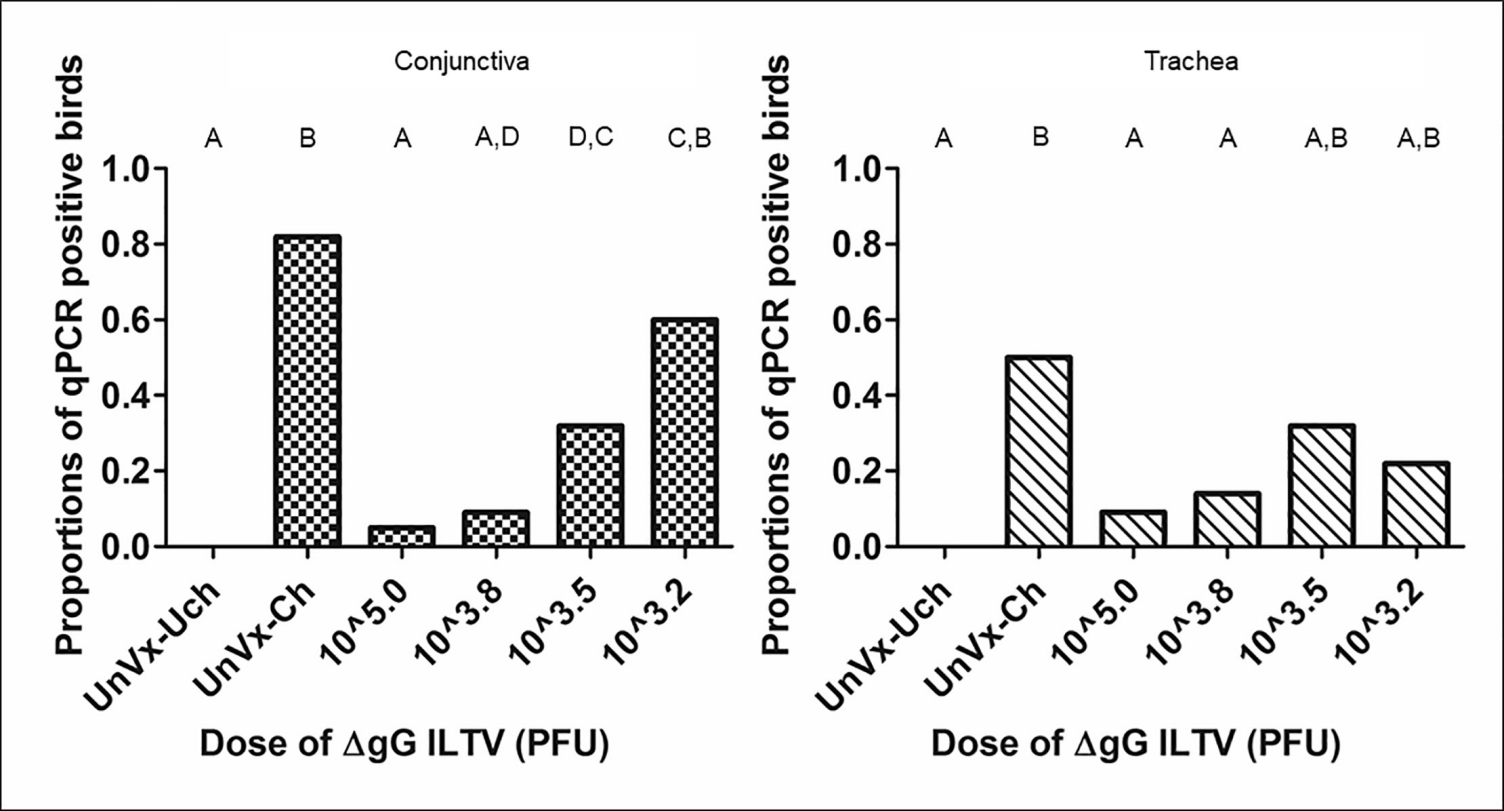
Bar graphs showing proportions of ILTV positive birds per group, as determined by qPCR on extracts from conjunctival or tracheal swabs of unvaccinated birds and birds vaccinated with different doses of ΔgG ILTV at day 7 after challenge with class 9 ILTV. Positivity was determined using the cut-off value of 100 genome copies number per reaction. UnVx-Uch = unvaccinated and unchallenged, UnVx-Ch = unvaccinated and challenged, PFU = plaque forming units. Values labelled with the same upper-case letter (A, B, C, D) were not significantly different (P > 0.05, Fisher’s exact test) in each panel (conjunctiva or trachea).

The proportions of birds that were positive after challenge for ILTV by qPCR are shown in Fig 2. A smaller proportion of birds that received the higher doses of the vaccine (10^3.8^ PFU or 10^5.0^ PFU) yielded ILTV positive conjunctival swabs (1/22 and 2/22 birds, respectively). The proportion of birds positive for ILTV DNA in the trachea after challenge was not significantly different between the negative control group and the groups that received the higher dose of the vaccine (10^3.8^ PFU or 10^5.0^ PFU), indicating a good level of protection in these vaccinated birds. In the group that received the lowest dose of vaccine, the proportion of ILTV positive birds (13/22) did not differ significantly from that of the positive control group (18/22). Fewer birds were positive for ILTV in the trachea compared to the conjunctiva (Fig 2).

### 3.3 Gross tracheal pathology and mortalities

Mortalities and gross tracheal pathology scores after challenge are shown in Table 4. No mortalities were seen in the birds vaccinated with the higher doses of vaccine (10^3.8^ PFU or 10^5.0^ PFU). There were four mortalities after challenge in each of the groups of birds that received the lower doses of vaccine (10^3.2^ PFU or 10^3.5^ PFU). These mortality rates were not significantly different from that seen in the positive control group (10/22).

**Table 4:** Mortality rates and gross pathological scores in unvaccinated birds and in birds vaccinated with different doses of ΔgG ILTV vaccine via eye-drop at day 7 after challenge.

Vaccine dose (PFU)	Challenge^‡^	N*	No. mortalities^†^^^^ (%)	Median pathology score (range)
None	None	10	0 (0) ^A, C^	0 (0–0) ^A^
None	Class 9^‡^	22	10 (45.5) ^B^	1.5 (0–3) ^B^
ΔgG ILTV (10^5.0^)	Class 9	22	0 (0) ^A^	0 (0–1) ^A^
ΔgG ILTV (10^3.8^)	Class 9	22	0 (0) ^A^	0 (0–1) ^A^
ΔgG ILTV (10^3.5^)	Class 9	22	4 (18.2) ^B, C^	1 (0–3) ^C^
ΔgG ILTV (10^3.2^)	Class 9	22	4 (18.2) ^B, C^	1 (0–3) ^C^

^‡^ Class 9 challenge = 10^3^ PFU of class 9 ILTV delivered via eye-drop and intra-tracheal inoculation

*N = number of birds in group

^†^ Values labelled with the same uppercase superscript letter in the same column were not significantly different (P > 0.05, Fishers exact test or Mann-Whitney test).

^^^ Birds that died or were euthanized due to severe clinical signs of disease following challenge

None of the birds in the negative control group had any tracheal pathology consistent with infection with ILTV. There was no significant difference in the tracheal pathology scores of the birds in the negative control group and those of the birds in the groups that received the two higher doses of vaccine (10^3.8^ PFU or 10^5.0^ PFU). The tracheal pathology scores of the groups of birds that received the lower doses of vaccine (10^3.2^ PFU or 10^3.5^ PFU) were significantly higher than the scores of the birds in the negative control group, but significantly lower than the scores of the birds in the positive control group.

## 4. Discussion

A dose of 10^3.8^ PFU was the lowest dose capable of providing a high level of protection against challenge with the class 9 ILTV. In this study, birds vaccinated with a dose of 10^3.8^ PFU were protected and did not develop clinical signs or tracheal pathology consistent with ILT. Replication of wild type virus was also greatly reduced in the conjunctival and tracheal mucosa of these birds but was not completely prevented. This is common for ILTV vaccines, which typically induce non-sterile immunity [12, 15, 17, 22, 23]. Increasing the vaccine dose to 10^5.0^ PFU did not significantly improve the level of protection compared to that induced by the minimum effective dose, whereas lower doses (10^3.2^ PFU or 10^3.5^ PFU) failed to fully protect the birds from clinical disease after challenge. Unprotected or incompletely protected birds may serve as reservoir of infection from which susceptible animals may become infected [5]. Thus, vaccines should not be used at a dose below the minimum effective dose. Using the lowest possible protective dose of vaccine (rather than a higher dose) is important for keeping vaccine production costs as low as possible, and thus the costs of vaccination as low as possible for producers.

Vaccination and challenge experiments are commonly performed to assess the level of protection induced by ILTV vaccines. Such studies are necessary because there are currently no easily measured correlates of protection (such as serum antibody levels) for ILTV infection, as cell mediated immunity [24, 25], rather than neutralising antibodies [26], are thought to protect against disease. Most ILTV vaccination and challenge studies assess levels of clinical protection by scoring clinical signs, tracheal pathology, and mortalities [2, 3, 12, 27]. Many also assess weight gain [15, 17, 27]. In this study, weight gain was not measured, as layer type birds do not typically have significant weight gains in short periods of time. Importantly, our study also assessed virus replication after challenge by measuring the viral genome concentration, as have some other vaccine efficacy studies [7, 12, 15, 17]. Vaccines that prevent clinical signs of disease but do not prevent replication of pathogens after challenge have been called ‘leaky’ or ‘imperfect’ vaccines and this is a common feature of ILTV vaccines [27–29]. Such vaccines may select for more virulent pathogens over time [30, 31] as has recently been demonstrated experimentally for Marek’s disease vaccines [30]. In our study, the concentrations of virus genomes in the conjunctival and tracheal mucosa were reduced to very low levels in birds that received high doses of the vaccine (10^3.8^ PFU or 10^5.0^ PFU) and only a small number of birds had detectable concentrations of ILTV DNA in the trachea or conjunctiva. These results suggest this vaccine induces significant protection against viral replication, although it should be noted that viral detection and quantification was assessed at one time point. The inclusion of additional time points would further clarify this observation, particularly earlier time points when viral titres are likely to be higher.

The results from this study demonstrate that Vaxsafe ILT induces a high level of protection when administered by eye-drop at a dose of 10^3.8^ PFU. This adds to the growing number of studies that have demonstrated the high level of safety and efficacy of the ΔgG ILTV strain when administered via different routes, using different challenge models [12, 13, 15, 16]. Glycoprotein G (gG) is a viral chemokine binding protein. Deletion of gG from the ILTV genome alters the immune response to ILTV infection [32], resulting in a more protective, cell-mediated immune response [20]. This vaccine also offers the potential to discriminate between vaccinated birds and birds infected with wildtype viruses using differential PCR [33] or ELISAs [19] in DIVA (differentiation of infected and vaccinated animals) control programmes. Although a number of gene-deleted ILTV strains have been shown to have *in vivo* phenotypes that may make them suitable for use as vaccines [12–16, 34–41], the ΔgG ILTV vaccine strain is the most extensively investigated of these deletion mutants, providing a high level of confidence in the performance of the vaccine under different conditions. This current study shows that the ΔgG ILTV vaccine strain is able to induce a high level of protection against a virulent recombinant field virus at a commercially feasible dose. These results lay the foundations for the application of a commercial vaccine product, offering poultry producers a new tool to help control ILTV.

## Abbreviations

ILTV: infectious laryngotracheitis virus
ILT: infectious laryngotracheitis
ΔgG ILTV: glycoprotein G-deficient ILTV
dpc: day post challenge
PFU: plaque forming unit
UnVx-Uch: unvaccinated and unchallenged
UnVx-Ch: unvaccinated and challenged
SPF: specific pathogen free
